# Supplementary material for: Challenges in observing transcription–translation for bottom-up synthetic biology
Source: QRB Discov. 2025 Jan 3;6:e5. doi: 10.1017/qrd.2024.27 (PMC11811876; doi:10.1017/qrd.2024.27)
Supplement: Bogatyr and Wuite supplementary material [file S2633289224000279sup001.docx]

# Supplementary materials

## PURE system composition

The list of PURE system components with their concentrations (Shimizu *et al.* 2005) is provided below.

**Translation factors:** 2.7 µM IF1, 0.40 µM IF2, 1.5 µM IF3, 0.26 cM EF-G, 0.92 µM EF-Tu, 0.66 µM EF-Ts, 0.25 µM RF1, 0.24 µM RF2, 0.17 µM RF3, 0.50 µM RRF, where IF - initiation factors, EF - elongation factors, RF - release (termination) factors, and RRF - ribosome recycling factor.

**Aminoacyl-tRNA synthetases:** 1900 U/ml AlaRS, 2500 U/ml ArgRS, 20 mg/ml AsnRS, 2500 U/ml AspRS, 630 U/ml CysRS, 1300 U/ml GlnRS, 1900 U/ml GluRS, 5000 U/ml GlyRS, 630 U/ml HisRS, 2500 U/ml IleRS, 3800 U/ml LeuRS, 3800 U/ml LysRS, 6300 U/ml MetRS, 1300 U/ml PheRS, 1300 U/ml ProRS, 1900 U/ml SerRS, 1300 U/ml ThrRS, 630 U/ml TrpRS, 630 U/ml TyrRS, 3100 U/ml ValRS.

**Other enzymes:** 4500 U/ml methionyl-tRNA transformylase, 1.2 µM ribosomes, 4.0 µg/ml creatine kinase, 3.0 µg/ml myokinase, 1.1 µg/ml nucleoside-diphosphate kinase, 2.0 units/ml pyrophosphatase, 10 µg/ml T7 RNA polymerase.

**Energy sources:** 2 mM ATP, 2 mM GTP, 1 mM CTP, 1 mM UTP, 20 mM creatine phosphate.

**Buffers:** 50 mM Hepes–KOH pH 7.6, 100 mM potassium glutamate, 13 mM magnesium acetate, 2 mM spermidine, 1 mM DTT.

**Other components:** 0.3 mM 20 amino acids, 10 mg/ml 10-formyl-5,6,7,8-tetrahydrofolic acid, 56 A260/ml tRNAmix (Roche).

Specific activities of aminoacyl-tRNA synthetases and methionyl-tRNA transformylase were measured using radioactive amino acids. One unit of activity was defined as the amount of enzyme that catalyzes the formation of 1 pmol of aminoacyl-tRNA in 1 min.

Notably, this composition is an exemplary one, as it was further adjusted and modified.

Shimizu, Y., Kanamori, T., & Ueda, T. (2005). Protein synthesis by pure translation systems. *Methods*, **36**(3), 299–304.
